# Unintentional Retention of Rectal Foreign Body Associated With Autoerotic Practices: A Case Report

**DOI:** 10.7759/cureus.79347

**Published:** 2025-02-20

**Authors:** Jekin J Sharon, Gokulesh Devannagoundanur Gurumurthy, Kamalraj M, Manisha M Puranikmath

**Affiliations:** 1 Institute of General Surgery, Madras Medical College, Chennai, IND

**Keywords:** anal foreign body insertion, dangerous sexual practices, foreign bodies, risky sexual behaviors, unintentionally retained foreign object

## Abstract

Rectal foreign bodies (RFBs) are now an increasing presentation in the emergency rooms. While they most often result from insertion for sexual gratification, other causes, such as body packing and criminal or accidental insertion, can also occur. Various foreign objects of different shapes, sizes, textures, and materials are encountered, sometimes with impaction, perforation, and peritonitis. Other challenges include delayed presentation and reluctance to provide required details due to social stigma. In our case, the patient presented with abdominopelvic pain and obstipation. Upon further questioning, he admitted inserting a sweet lime fruit with sexual intent on the previous day. Digital rectal examination revealed a hard, round mass measuring 7 × 7 cm, located 4 cm from the anal verge. There were no signs of perforation or peritonitis; an X-ray confirmed the same. Since the anal sphincter was lax, a transanal extraction was attempted at the emergency room. The patient was anxious and failed to relax his anal sphincter completely. Hence, he was taken to the operating room. Under intravenous sedation and perianal block, a well-lubricated proctoscope was introduced, and a foreign body was incised to facilitate the grasping of the outer peel. The foreign body was then removed in pieces with ease. Postoperative proctosigmoidoscopy was normal, and the patient was discharged after psychiatric assessment and counseling. Hence, a systematic approach is recommended in diagnosing and managing RFBs. Psychiatric assessment and follow-up are also crucial in these patients.

## Introduction

The incidence of rectal foreign bodies (RFBs) is rising in the emergency departments, especially in the urban population. Most of them are men in their 40s [1]. The insertion of foreign bodies into the rectum can be voluntary, such as for sexual gratification, body packing, or other purposes. Sometimes, involuntary insertion of RFBs is also possible (accidental/criminal). However, concerning RFBs, anal autoeroticism is the most common cause, with glass bottles being the most frequently encountered objects (42.2%) [2]. RFBs are difficult to diagnose due to the hesitancy of the patient to reveal the incident and delayed presentation due to the social stigma attached. The variety of RFBs encountered and the broad spectrum of injuries add to the complexity [3]. The complications associated (impaction, perforation, and peritonitis) mandate a systematic workup in these cases for effective treatment [1,2]. Here, we present a case of RFB and its management and a brief literature review.

## Case presentation

A 50-year-old man presented with complaints of lower abdominopelvic pain and obstipation for the past one day. There was no history of fever, vomiting, or abdominal distension. He had no history suggestive of renal/bladder calculi or cystitis. Upon further questioning, he admitted to the rectal insertion of a sweet lime the day before. He was known to insert foreign objects rectally for anal autoeroticism. He had initially begun inserting small objects that he removed with ease. This was the first time he had presented to the emergency room with multiple failed attempts at removal. He identified himself as a heterosexual and denied any history of anal receptive intercourse. He had a history of incontinence to flatus. There was no history of rectal bleeding.

**Figure 1:**
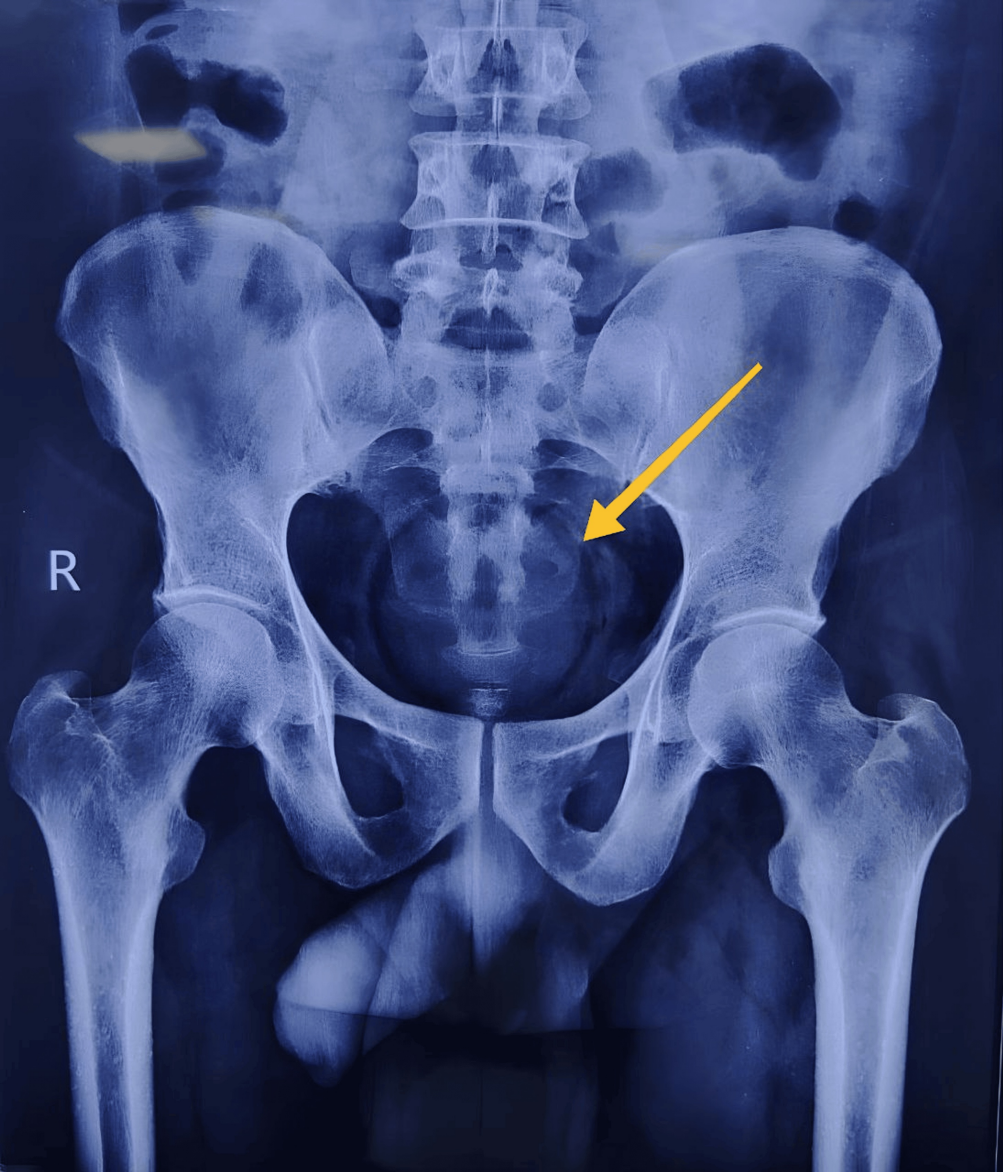
X-ray abdomen erect, anteroposterior view showing the RFB (yellow arrow) in the lesser pelvis RFB: rectal foreign body

On examination, vitals were stable, and the abdomen was soft without any palpable mass or tenderness. Bowel sounds were present. External genitalia were normal. The lateral buttock traction test was positive. Digital rectal examination revealed a hard hemispherical mass of about 7 × 7 cm located 4 cm from the anal verge. The tone of the anal sphincter was decreased, and fecal staining was absent. Proctoscopy revealed a normal mucosa without tears or ischemic compromise, and the RFB (sweet lime) was visualized with ease. An erect X-ray of the abdomen revealed a spherical RFB within the lesser pelvis; there were no signs of intestinal obstruction (multiple air-fluid levels) or perforation (air under the diaphragm) (Figure 1).

**Figure 2:**
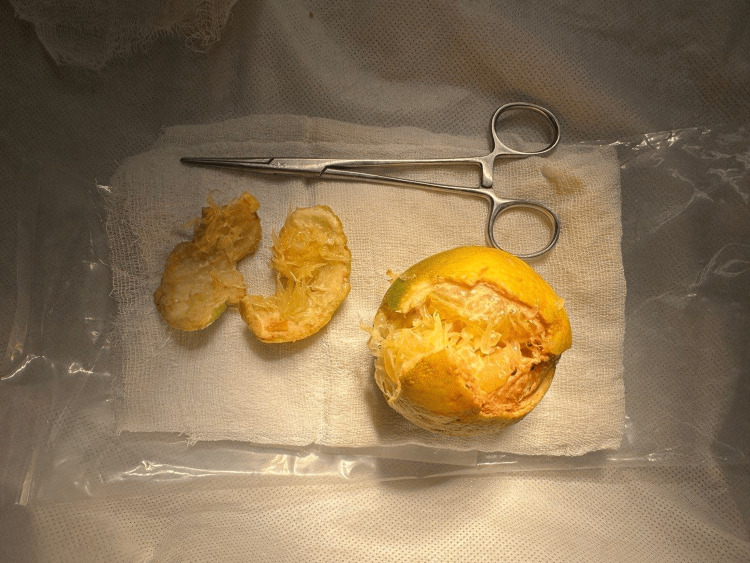
Foreign body (sweet lime) retrieved transanally from the rectum. The foreign body was incised to facilitate grasping and to break the air seal Forceps placed for scale

Since the tone of the anal sphincter was decreased, simple hand maneuvering was tried along with forceps (for traction) and simultaneous suprapubic pressure. However, the efforts were unsuccessful as the patient was anxious and failed to cooperate. Hence, he was taken to the operating room and placed in a lithotomy position with reverse Trendelenburg angulation. He was given intravenous sedation and a perianal block. The parts were painted and draped, followed by an injection of local anesthetic solution into the ischiorectal fossa at the level of the levator ani muscles. The needle was inserted about 3 cm from the anal verge through the anococcygeal ligament, and the solution was injected while withdrawing the needle. Then, the needle was angulated 45° anterolaterally, and the solution was injected while withdrawing the needle. The same procedure was repeated in the anterior perianal region. Finally, two injections were made lateral to the anal opening, thereby achieving complete anesthesia of the perianal skin and anal canal along with relaxation of the sphincters. A proctoscope was introduced, and RFB was visualized. The RFB (sweet lime) was incised to facilitate grasping the outer peel with Allis tissue forceps. Later on, Langenbeck retractors were used, and the RFB was removed in three parts (Figure 2, Video 1).

**Video 1:** Transanal retrieval of the foreign body from the rectum

The postoperative period was uneventful. A sigmoidoscopy done on the next day showed only mild mucosal hyperemia. The patient was discharged the next day and was advised to follow up further in the psychiatry outpatient department. The psychiatric evaluation ruled out psychopathological causes of RFBs, such as sexual paraphilia, psychotic disorders, depressive disorders, factitious disorders, and malingering. It was concluded that the patient had performed the act for sexual gratification. At the one-month follow-up visit, the patient reported improvement in continence following regular Kegel exercises.

## Discussion

RFBs are a proctological emergency. Even though the incidence of RFBs is increasing, the exact numbers are yet to be calculated as most of the available data are in the form of case reports or retrospective case series, most of which are from the Western world [1]. We have added one such case encountered at our tertiary care center in Chennai (South India) to the existing literature. The list of objects encountered is exhaustive and includes household objects like toothbrushes, broomstick handles, fruits and vegetables, cosmetics (lip balms and deodorant bottles), cans or bottles, batteries, torches, light bulbs, children's toys, sex toys (dildos and vibrators), etc. [3,4]. In decreasing order of frequency, the reasons for RFBs include anal autoeroticism, concealment, seeking attention, accidental occurrences, criminal/assault, and alleviating constipation (especially noted in the elderly) [4].

**Figure 3:**
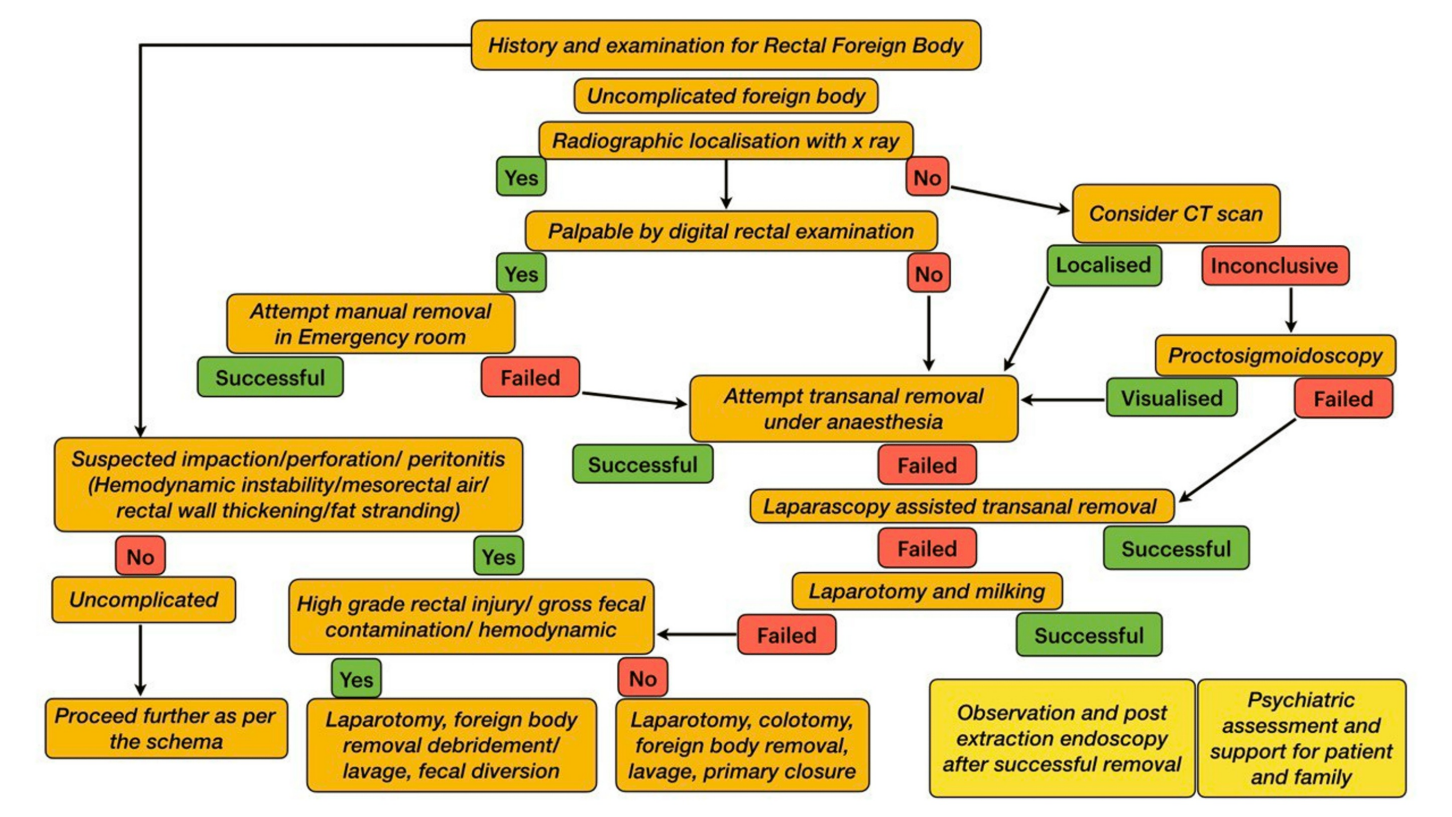
Our proposed algorithm for stepwise evaluation and management of RFB CT: computed tomography; RFB: rectal foreign body

If the former fails, RFBs should be managed using a stepwise approach, progressing from the least invasive to more complex procedures. The algorithm shown in Figure 3 can be followed to remove RFBs [3,5]. Further studies are needed to validate our proposed algorithm.

The first step is to determine if there is a perforation and if the patient is stable in cases with high suspicion of perforation. Laboratory investigations may reveal an elevated total leukocyte count/acidosis if there is perforation (intraperitoneal/extraperitoneal) or mucosal ischemia from pressure necrosis, but radiological investigations are more important. Peritonitis is highly likely if the perforation is above the level of peritoneal reflection and is not contained [5]. Hypotension, tachycardia, severe abdominopelvic pain, and fever indicate perforation or peritonitis. In such situations, immediate resuscitation with intravenous fluids and broad-spectrum antibiotics is necessary. If the patient remains stable despite perforation (often extraperitoneal in these cases), computed tomography may reveal rectal thickening, fat stranding, and air in the mesorectum [1,6].

Most RFBs (60%-75%) can be removed by a transanal approach, which requires adequate analgesia and sedation to ensure patient cooperation and sphincter relaxation. Lithotomy position with reverse Trendelenburg angulation is ideal [6]. A perianal block is considered superior for these cases. The technique described by Nyström et al. was used in our case with good results [7]. Suction devices, vaginal spatulas, vulsellum forceps, and obstetric outlet forceps can be used to aid removal. Placing a Foley catheter alongside the RFB can aid removal in two ways: by breaking the air seal and by facilitating traction [8]. The transanal approach is contraindicated if there is peritonitis secondary to perforation. A sharp or fragile RFB is a relative contraindication to this approach [9]. Sharp objects and objects not within the reach of hand/tools (like graspers) or objects not visualized by a proctoscope necessitate endoscopic removal. A rigid sigmoidoscope is more often helpful than a flexible sigmoidoscope. Once the object is visualized, gentle insufflation and polypectomy snare or biopsy forceps can be used to safely remove the RFBs [1,9,10]. Laparoscopic-assisted foreign body extraction can be done by “milking” the RFB distally to facilitate transanal removal [11]. If it fails, colostomy may be necessary. Lake et al. reported that laparotomy was required only in less than 10% of the patients in their series [6]. It is used as a last resort after failed attempts of transanal removal under anesthesia or the presence of peritonitis requiring lavage, fecal diversion, etc. [8,10,12]. A Hartmann procedure is ideal in the presence of large perforation, pressure necrosis (due to ischemia), and gross fecal contamination [8,12]. A small, fresh perforation with limited peritoneal contamination is feasible for primary repair. Body packers represent a distinct subset of cases in which surgical removal of RFBs is mandatory, particularly when the illicit substance is contained in fragile packaging. This approach is essential to minimize the risk of substance spillage and subsequent systemic toxicity [13].

Postextraction proctosigmoidoscopy has been recommended in all cases to visualize the degree of mucosal damage, if any. Lake et al. have noted that if there is no injury at presentation, it is unlikely to find one after the removal of RFB [6]. Mental health and social aspects of RFB are as important as surgical management. The health care provider must provide emotional support, treat the patient with respect, and ensure privacy from curious hospital staff not involved in the care of that patient. Hence, postprocedural counseling and a psychiatric assessment are imperative to aid mental well-being [1,8].

## Conclusions

RFBs represent a critical proctological emergency that requires a careful, systematic approach for diagnosis, management, and prevention of potential complications. Beyond immediate surgical or procedural management, a holistic care framework is indispensable to address the multifaceted psychosocial dimensions often underlying RFB presentations. This approach encompasses comprehensive education on safe sexual practices, along with psychiatric assessment and support for both the patient and their family. Crucially, the prevention of recurrent episodes hinges on sustained psychiatric follow-up and tailored behavioral interventions. We emphasize adopting this comprehensive strategy, which combines urgent clinical management with mental health and social support, as a standard for RFB cases. Such multidisciplinary alignment underscores the importance of addressing both anatomical and psychosocial vulnerabilities in this complex clinical scenario.

In this case, successfully implementing a perianal nerve block with intravenous sedation provided adequate anesthesia and analgesia, effectively circumventing the need for spinal anesthesia and its inherent risks, such as hemodynamic instability, postdural puncture headache, and urinary retention. This approach not only minimized procedural invasiveness but also reduced recovery time and enhanced patient comfort. Based on these outcomes, we advocate for the consideration of perianal block with intravenous sedation as a safe, practical, and patient-centered alternative to spinal anesthesia in select clinical scenarios requiring perineal or anorectal interventions.
